# Rationally combining anti-VEGF therapy with radiation in NF2 schwannoma

**Published:** 2016

**Authors:** Na Zhang, Xing Gao, Yingchao Zhao, Meenal Datta, Pinan Liu, Lei Xu

**Affiliations:** 1 Department of Radiation Oncology, Massachusetts General Hospital and Harvard Medical School, Boston, Massachusetts 02114; 2 Department of Otolaryngology Head and Neck Surgery, Beijing TongRen Hospital, Capital Medical University, Beijing China, 100730; 3 Department of Oral and Maxillofacial Surgery, Xiangya Hospital, Central South University, Changsha, Hunan 410008, China; 4 Cancer Center, Union Hospital, Tongji Medical College, Huazhong University of Science and Technology, Wuhan, Hubei 430023, China; 5 Department of Chemical and Biological Engineering, Tufts University, Medford, MA 02155, USA; 6 Neural Reconstructional Department, Beijing Neurosurgical Institute, Capital Medical University, Beijing, China, 100050

**Keywords:** Neurofibromatosis type 2 (NF2), Anti-VEGF therapy, Radiation therapy, Schwannoma, Tumor

## Abstract

Neurofibromatosis type 2 is characterized by bilateral vestibular schwannomas, which are benign tumors that originate from the nerve sheath and damage the nerve as they grow, causing neurological dysfunction such as hearing loss. Current standard radiation therapy can further augment hearing loss by inducing local damage to mature nerve tissue. Treatment with bevacizumab, a Vascular Endothelial Growth Factor (VEGF)-specific antibody, is associated with tumor control and hearing improvement in NF2 patients; however, its effect is not durable and its mechanism of action on improving nerve function is unknown. Anti-VEGF treatment can normalize the tumor vasculature, improving vessel perfusion and delivery of oxygen. It is known that oxygen is a potent radiosensitizer; therefore, combining anti-VEGF treatment with radiation therapy can achieve better tumor control and allow for the use of lower radiation doses, thus minimizing treatment-related neurological toxicity.

## Current challenges in NF2 therapy

Neurofibromatosis type 2 (NF2) is a dominantly inherited genetic condition with a birth prevalence of 1 in 25,000^1^. NF2 is characterized by bilateral vestibular schwannomas (VS), which are benign tumors composed of neoplastic Schwann cells that arise from the eighth cranial nerve, which transmits hearing and balance information from the ears to the brain. Although these vestibular schwannomas grow slowly, they usually lead to a significant or total hearing loss by young adulthood or middle age. The tumors can also compresses the brain stem, leading to headaches, difficulty swallowing, and other serious neurologic symptoms^2^. Standard approaches for the treatment of growing VS include surgical resection and radiation therapy (RT). While these tumors can be successfully removed or destroyed with surgery and radiation treatment, paradoxically, these therapeutic approaches can also cause hearing loss. For patients with sporadic VS who do not have NF2, RT is associated with long-term tumor control rates exceeding 95%. However, hearing preservation rates after radiation range from 50-80%^3,4^. Post-RT outcomes for patients with NF2 are inferior to those for sporadic patients, with short-term local tumor control rates around 80-85% and hearing preservation rates less than 50%^3^. Thus, the identification of a novel adjunct therapy to enhance radiosensitivity while minimizing toxicity-related hearing loss in VS is urgently needed.

## Recent advances in targeted therapy for NF2

Several previous investigations have suggested that – unlike other benign tumors – vestibular schwannomas, like malignant tumors, are able to induce the formation of new blood vessels. Vascular endothelial growth factor (VEGF) and its receptors (VEGFRs) are expressed in VS, and VEGF expression level positively correlates with schwannoma growth rate^5-7^. A 2009 New England Journal of Medicine study led by Scott Plotkin, MD, PhD, reported that treatment with bevacizumab, a humanized monoclonal antibody that specifically neutralizes VEGF-A, was associated with a reduction in the volume of most growing VS. More importantly, bevacizumab treatment improved hearing in 57% patients^7^. Limitations of anti-VEGF treatment – the fact that not all patients responded, that hearing improvement was often transient and the effect of anti-VEGF on nerve function is not known, and that some patients could not tolerate long-term bevacizumab treatment – indicated the need to better understand the mechanisms of anti-angiogenic therapy on the function of tumor-bearing nerves.

## Rationally combining anti-VEGF therapy with radiation

Anti-VEGF agents were originally developed to block tumor growth by inhibiting blood vessel formation^8,9^. Bevacizumab has failed to improve survival benefit as a monotherapy in a number of tumors, but has been shown to confer survival benefit in combination with chemotherapy^9^. A potential explanation for the success of combined therapies is that bevacizumab “normalizes” the abnormal vasculature of tumors. It has been shown in many preclinical and clinical studies that anti-angiogenic therapy prunes tumor vessels and reverts the abnormal structure and function of the remaining vasculature toward a more normal state, abrogating its deleterious effects on the tumor microenvironment^10^. However, the normalization effect is transient – leading to a “normalization window” – during which the resulting vasculature is more normal, characterized by increased blood flow and improved delivery of concurrently administered anti-cancer drugs, as well as oxygen^9^. The addition of anti-angiogenic therapy to chemotherapy is now standard treatment for a variety of metastatic cancers including colorectal cancer and nonsquamous cell lung cancer.

Given the role of tissue oxygenation in tumor response to radiation, as well as the potential protective role of VEGF against endothelial cell apoptosis in response to radiation, several preclinical studies have demonstrated that anti-angiogenic treatment potentiates the effects of radiation therapy against various solid tumors established from cell lines in xenograft models (Table 1). To date, early-phase clinical trials have demonstrated promising response rates and tolerability of combining bevacizumab with radiation for the local control of various primary, recurrent, and metastatic tumors (Table 2)^11-14^. These studies have found that some additional toxicities occur with the combination of bevacizumab, but common toxicities such as hypertension and proteinuria are generally easily managed while severe toxicities are rare. However, the reported response rate has varied, indicating the need for a rational pre-selection of patients for this combination treatment, as well as prospectively validated biomarkers of response^12,15-19^.

## Combining anti-VEGF treatment with radiation therapy achieves better tumor control and minimizes radiation-related neurological damage in NF2-related schwannoma models

Although there are many reports in the context of malignant cancers, little is known of the effect of combined anti-VEGF treatment with radiation therapy in benign tumors. Recent studies led by Lei Xu, MD, PhD, at Massachusetts General Hospital report that combining anti-VEGF treatment with radiation therapy improves the effectiveness of radiation treatment in NF2 related vestibular schwannoma models, and that the combination allows the use of a lower radiation dose to achieve the same degree of tumor control as a higher radiation dose without anti-VEGF therapy^20^. As a step further, this study shows that combining anti-VEGF treatment with radiation improves neurologic function by i) reducing the dose of radiation therapy and minimizing treatment-associated adverse effects, and ii) alleviating tissue edema, which may further improve neurologic function by decreasing muscle atrophy and increasing nerve regeneration^20^. This study provides compelling rationale and paves the road for testing combined anti-VEGF therapy with RT in NF2 related vestibular schwannomas. In preparation for future clinical studies with combined anti-angiogenic and RT, clinical studies of the therapeutic effects of anti-VEGF treatment on radiation-induced nerve damage need to be thoroughly examined. Furthermore, characterization of the schwannoma vasculature (including relative schwannoma vessel size and permeability, tumor contrast enhancement, edema-associated parameters from MRI), and biomarkers studies are needed to fully elucidate the normalization effect of bevacizumab in NF2 patients, and are needed before clinical studies with combined anti-angiogenic and RT can be designed.

## Figures and Tables

**Table 1:** Preclinical studies of combined anti-angiogenic and radiation therapy.

Tumor	Cell line	Result	Reference
NF2 Schwannoma	HEI193 NF2^−/−^	Enhanced tumor inhibition Decreased dose of radiation Improved neurological function	(20)
Glioblastoma	U87	Enhanced tumor inhibition Decreased dose of radiation	(21-25)
	U251	Enhanced tumor inhibition Decreased dose of radiation	(24)
Head and neck cancer	SCC1	Enhanced tumor inhibition	(26)
Colorectal cancer	LS174T	Enhanced tumor inhibition Reduced radioresistance	(21)
	SW480	Enhanced tumor inhibition	(27)
Ovarian carcinoma	MA148	Enhanced tumor inhibition	(28)
Melanoma	B16F10	Enhanced tumor inhibition	(28)
Lung cancer	54A	Decreased dose of radiation	(23)
	H226	Enhanced tumor inhibition	(26)
	A549	Enhanced tumor inhibition Decreased dose of radiation	(29)

**Table 2:** Clinical trials of combined anti-angiogenic and radiation therapy.

Tumor type	Phase	Number of Patients	Radiation dose /Fraction	Bevacizumab Dose	Outcome		Reference
CNS Tumor							
nGBM	III	978	60Gy/2Gy	10mg/kg	Median PFS: 10.7 months	Median OS: 15.7 months	(30)
nGBM	III	921	60Gy/2Gy	10mg/kg	Median PFS: 10.6 months	Median OS: 16.8 months	(31)
nGBM	II	70	60 Gy/30	10 mg/kg	PFS: 19.6%	OS: 13.6%	(32)
nGBM	II	75	59.4 Gy/33	10 mg/kg	PFS: 21.2%	OS: 14.2%	(33)
nGBM	II	125	50.4Gy/1.8Gy	10mg/kg	Median PFS: 13.8 months	1-year PFS: 63.1%	(34)
nGBM	II	68	60Gy/2Gy	10mg/kg	Median PFS: 11.3 months	Median OS: 13.9 months	(35)
nGBM	II	48	60Gy	10mg/kg	Median PFS: 9.2 months	Median OS: 13.2 months	(36)
rGBM	I	25	30 Gy/5	10 mg/kg	PFS: 12.5-16.5%	OS: 7.3-7.5%	(37)
rGBM	I/II	15	25Gy/5	10mg/kg	Median PFS: 3.9 months	Median OS: 14.4 months	(38)
Head and neck cancer							
HNSCC	II	30	56-70Gy/35	5 mg/kg	3-year PFS: 61.7%	3-year OS: 68.2%	(39)
HNSCC	0	29	9.5-71.5Gy/1.25Gy	10mg/kg	3-year PFS: 82%	3-year OS: 86%	(40)
HNSCC	II	42	70Gy/2.12Gy	15mg/kg	2-year PFS: 75.9%	2-year OS: 88%	(41)
HNSCC	II	78	70Gy/35	15mg/kg	2-year PFS: 75%	2-year OS: 88%	(42)
HNSCC	II	30	70Gy/35	15mg/kg	2-year PFS: 88.5%	2-year OS: 92.8%	(43)
Nasopharyngeal	II	46	70Gy/33	15mg/kg	2-year PFS: 74.7%	2-year OS: 90.9%	(44)
Gastrointestinal cancer							
Esophagus	II	62	45Gy/1.8Gy	15mg/kg	pCR: 29%		(45)
Colorectal	II	32	45Gy/1.8Gy	5mg/kg	pCR: 25%	4-year OS: 91%	(46)
Rectal	II	66	50.4Gy/28	5mg/kg	1-year DFS: 85% 2-year DFS: 97%		(18)
Rectal	I	11	50.4 Gy/28	10-15 mg/kg	pCR: 18%		(14)
Rectal	I/II	32	50.4 Gy/28	5-10 mg/kg	pCR: 16%		(13)
Rectal	II	25	50.4 Gy/28	5 mg/kg	pCR: 32%		(15)
Rectal	II	61	50.4 Gy/28	5 mg/kg	pCR: 13%		(47)
Rectal	II	42	50.4 Gy/28	5 mg/kg	pCR: 18%		(12)
Rectal	II	59	45Gy/1.8Gy	5 mg/kg	pCR: 36%		(48)
Rectal	II	91	45Gy/25	5 mg/kg	pCR: 23.7%		(49)
Gynecological cancer							
Cervical	II	60	45Gy/25	10mg/kg	No treatment-related SAEs		(50)
Endometrial	II	34	45Gy/25	5mg/kg	2-year PFS: 79.1%	2- year OS: 96.7%	(51)
Endometrial	II	15	45Gy/25	10mg/kg	1-/3-year PFS: 80%/67%	1-/3-year OS: 93%/80%	(52)
Ovarian		4			1-/3-year PFS: 80%/40%	1-/3-year OS: 100%/60%	

Abbreviations: CNS: central nervous system, nGBM: newly diagnosed glioblastoma, rGBM: recurrent GBM, MG: malignant glioma, HNSCC: head and neck squamous cell carcinoma, RT: radiation therapy, PFS: progression-free survival, OS: overall survival, SAEs: serious adverse events, pCR: pathologic complete response, SRS: stereotactic radiosurgery
